# 

**Published:** 1910-07-16

**Authors:** 


### BOOKS RECEIVED.

Wm. Rider and Sons.
"Power of Self-Suggestion." By Samuel McComb, D.D.
"How to Keep Fit." A. T. Schofield.

J. & A. Churchill.
"Practice of Midwifery." By Galabin and Blacker.

Methuen & Co.
"Laws of Heredity." By G. A. Reid.

### Appointments.

Dr. J. Campbell McClure has been appointed clinical assistant to the West End Hospital for Diseases of the Nervous System, Paralysis, and Epilepsy. Dr. McClure is M.D. and C.M. of the University of Glasgow, and before coming to London in April was chief assistant to the Professor of Medicine in that University and physician to out-patients at the Western Infirmary and Sick Children's Hospital at Glasgow.

Dr. T. R. Elliott has been appointed assistant physician to University College Hospital. He graduated M.D.Cantab. in 1908 and M.R.C.P. in the present year, has been a Fellow of Clare College, Cambridge, a scholar of Trinity, and has published many physiological researches, chiefly upon the innervation of the bladder and rectum. Dr. Elliott won the Raymond Horton-Smith prize of his University, and various research studentships.

### COMING EVENTS OF THE WEEK.

[Communications for this column must be sent to 29 Southampton Street, Strand, before noon on Wednesday.]

Saturday, July 16.—Royal Sanitary Institute. Provincial Sessional Meeting. Cambridge, 11 a.m.

Sunday, July 17.—Visiting Day at the Hospitals.

Tuesday, July 19.—Prince Christian presides at Guildhall Meeting, Windsor, to discuss plans for a permanent memorial to the late King, 4 p.m.

West London Hospital: Dr. Elliot. The Acute Abdomen. Lecture. 5 p.m.

Thursday, July 21.—Polyclinic, Chenies Street. Sir Jonathan Hutchinson's Museum Demonstration, 4 p.m.

Friday, July 22.—Royal Sanitary Institute. Provincial Sessional Meeting, Torquay, 11 a.m.

The following gentlemen have been appointed representatives on the Wigan and District Smallpox Joint Hospital Committee: Mr. J. Lucas being appointed for Aspull, Mr. A. Helme for Blackrod, Mr. H. Mather for Billinge, Mr. J. Anders for Upholland, Mr. C. Hartley for Orrell, Mr. A. Lymn for Ince, Mr. J. Mitchell for Ashton, and Mr. J. Belshaw, Mr. G. Short, Mr. C. Elliott, and Mr. J. Catterall West for Hindley. Mr. Lymn has been re-elected chairman.

### Gifts and Bequests.

Colonel John Edward Cutler, of Sheffield, left £2,000 to the Jessop Hospital for Women, and has left £100 each to six charitable institutions in Sheffield.

Among the latest contributions received for King Edward's Hospital Fund for London is £1,500 from the Drapers' Company, being the half-yearly payment of their annual subscription of £3,000.

Princess Dolgorouki has sent £150 to the secretary of the British Home and Hospital for Incurables, Streatham, S.W., being the proceeds of a concert held at "Nashdom" for the benefit of this institution.

Mr. Frederick Locock, of Leamington Spa, has bequeathed £400 to the churchwardens of the Sevenoaks Parish Church upon trust for such parochial charities as they may think fit, conditional upon their keeping in order his grave and monument, and failing the observance of this condition the bequest to revert to Queen Charlotte's Lying-in Hospital.

Prince Francis of Teck has received the following in response to his appeal on behalf of the Middlesex Hospital: Messrs. N. M. Rothschild and Sons, £500; Messrs. Barnato Brothers, £500; Messrs. Antony Gibbs and Son, £100; the Duchess of Marlborough, £100; Anonymous, £250; Mr. Paul Nelka, £100; Sir Edward Green, Bart., £100; Mr. C. Birch Crisp, £100; Messrs. Davies and Son, £105; Messrs. Debenham and Co., £105; Mr. F. Eckstein, £100; Anonymous, £500; Mr. Adolph Lewisohn, £150; Messrs. W. and A. Gilbey, £105; Mr. Samuel Whitbread, £100; Anonymous, £100; the Duke of Bedford, £100; the Governors and Company of the Bank of England, £105; Lady Wantage, £100; Messrs. Crosse and Blackwell, £105; and Messrs. Hunt and Roskell, £105.

Among recent contributions towards clearing off the debt of £17,000 on the Royal National Orthopædic Hospital, Great Portland Street, are £100 from Mrs. James Packe; £52 10s. from the Fishmongers' Company; £50 from Lord Dysart, Mrs. Sarah Walker Bubb, and Mr. Otto Beit; £45 from Miss A. Thomas; £27 12s. 4d. from Mr. H. H. Everett; £26 5s. from the Duke of Marlborough and Mr. Howard Morley; £25 from Colonel Hankey and Mr. E. Tate; and £21 from Sir Richard Martin.

### Jottings.

The King has become patron of St. Mary's Hospital and the Great Northern Central Hospital.

Mr. David Beatty, of Brooksby Hall, has very generously expressed a wish to maintain a cot in the ophthalmic wards of the Leicester Infirmary, to be called the "Peter" cot.

A town's meeting at Salford last week, presided over by the Mayor, decided to establish a King Edward Memorial fund. The proceeds are to be devoted to the Salford Royal Hospital. The motion was proposed by Sir William Mather and seconded by the Bishop of Manchester. Subscriptions amounting to nearly £4,000 were announced at the meeting.

Under the immediate patronage of his Serene Highness Prince Francis of Teck and a very distinguished *comite d'honneur*, Mr. Vaughan Grey will give a special *matinée* in aid of the Middlesex Hospital Fund at the Boudoir Theatre, Pembroke Gardens, W., on Thursday, July 28, at 3.30 p.m., for which a particularly interesting programme is announced.

The new home for nurses, the Toronto Hospital for Incurables, was opened recently. The present capacity of the hospital is 140, and a new wing providing for 90 additional beds is nearing completion.

St. Louis, in the United States, is to have a hospital department organised entirely separate from the Health Department. It is hoped in making this change to terminate political control of hospitals and attract to the institution a higher class of physicians than it has been possible to secure under former conditions.

The People's Hospital, of New York, was opened on June 21 last. The building is fireproof throughout and has accommodation for 50 patients. Most of the equipment was given, among the donors being the Emperor of Austria, who gave an operating table. An innovation on the medical staff is a "visiting gastro-enterologist."

Lord Rothschild laid the foundation-stone last week of a new hospital for Ealing, as a memorial to King Edward, and it is, by special permission, to be called the Ealing King Edward Memorial Hospital. The cost of the scheme is £17,500, towards which the sum of £12,612 has been raised, including £3,000 from the King Edward Hospital Fund.

Prince Alexander of Teck, the president, inspected the Royal Sea-Bathing Hospital at Margate last week, accompanied by the treasurer, Lord Biddulph, Mr. Newton Mappin, vice-president, and Hon. Alexander Yorke, C.V.O., Lord Forester and other directors, Sir Henry Lennard, chairman of the committee of management, and the secretary.

The Leicester Matinee Committee has been able to hand over £115 9s. 3d. to the funds of the Leicester Infirmary and Children's Hospital. The thanks of the Board have been conveyed to Mr. Oswald Stoll, and to the local committee, of whom Mr. H. H. Wildt, was chairman, Mr. Arthur Baum treasurer, and Mrs. J. H. Ward and Mr. C. Finch Hatton, hon. secretaries.

From a Saturday fête organised by the Broughton Astley (Leicester) Social Club a sum of £25 18s. 6d. has been sent to the Leicester Infirmary. The success of the fête was unfortunately marred by wet weather, but it says much for the spirit and enthusiasm in which the promoters (led by Mr. W. F. Dear) went to work that they reached within 1s. 9d. of their record contribution. It is worthy of note that the population of the village is only just over 1,100.

Sir Frederick Treves and an architect having reported, as the result of a special inspection, that the Chichester and West Sussex Infirmary buildings are inadequate for the increased work and for modern scientific requirements, it has been decided to make large alterations and additions, estimated to cost £20,000 to £24,000, which it is proposed to form as a memorial to King Edward VII. Towards this amount Mr. W. D. James, of West Dean Park (who is a member of the Management Committee) has promised £10,000.

The Chicago Hospital Day Association has recently been incorporated. Its object is to promote systematic charitable giving for the support of hospitals in Cook County working under church auspices. Methodists, Baptists, Roman Catholics, and Presbyterians have thus far taken out memberships. Seventy-five women from each church constitute the active members, and an advisory board of business men is provided for. The working plans are similar to those of the New York Hospital Saturday and Sunday, and the receipts are divided in proportion to the free work done.

